# Using composite system index to identify China’s ecological and socio-economic transition zone

**DOI:** 10.3389/fpls.2022.1057271

**Published:** 2022-11-22

**Authors:** Hao Zhang, Fei Liu, Jinying Zhang

**Affiliations:** ^1^ Institute of Mountain Hazards and Environment, Chinese Academy of Sciences, Chengdu, China; ^2^ Shandong Provincial Institute of Land Surveying and Mapping, Jinan, China

**Keywords:** ecotone, coupled system, agricultural production, human and natural interaction, transition zone

## Abstract

Regions with synthetic geographical gradients tend to exhibit distinct ecological transitions. As a compound ecosystem, transition zone can provide a basis for decision-making in the sustainable ecological management by investigating its boundary and complexity. To determine the characteristics of the transition zone where natural ecological and socio-economic factors interact, a conceptual framework and a quantitative identification method for the ecotone of coupled human and natural systems have been proposed. The composite system index can be used to ascertain the coupling intensity, coupling direction, and ecological transition of the system. Taking China as an example, this study showed evidence of the existence of a tremendous amount of ecological and socio-economic transition zone (complex coupled areas) between the east and west of China, and sporadic ecotone in other regions of the country. This transition zone accounted for about 1/4 of China’s land surface area, and had a fragile environment that faced challenges of environmental protection and economic development. In the area across the Hu Line, human and natural factors jointly explain a low proportion of the variance in ecological and socio-economic transition zone (the complexity of coupled systems, with 62.01% of unexplained proportion higher than that in other regions). In this region, the topographic position index was the critical element associated with the transition zone, and accounted for nearly 20% of the variation of composite system index. The discovery and characterization of the ecological and socio-economic transition zone is crucial for understanding its uncertainty and diversity and the complex of coupled ecosystems.

## 1 Introduction

Almost all mountainous countries have diversified and complex ecosystem, especially large countries like China (Zhang et al., 2022). Due to increasing human activity, the dynamics and complexity of interactions between human systems and natural systems are aggravated. Therefore, the ecosystems are increasingly characterized by uncertainty, variability (slow or rapid changes), interactivity, and transition characteristics (Vizzari et al., 2018; Deng et al., 2020). Ecotones are created when there are significant interactions between humans and the environment (Liu et al., 2021a); for example, urban–rural ecotones (Arnaiz-Schmitz et al., 2018; Yang et al., 2018), the agro-pastoral ecotones (Qiao et al., 2018; Chen et al., 2021), and the ecological ecotones (Williams et al., 2009; Blackwood et al., 2013; Yu et al., 2015). These regions have highly sensitive and fragile ecosystems, where conflicts between conservation and development create challenging socioeconomic and environmental issues (Antonelli et al., 2018).

To address the challenges posed by global changes, it is becoming increasingly clear that a coupled perspective of human and natural systems is crucial (Rammer and Seidl, 2015). The integrated functional system composed of human and natural environments is called the coupled human and natural ecosystem (Liu et al., 2007). Researchers have begun to pay increasing attention to the interdisciplinary field of synthetic ecosystem research, and the interactions between humans and nature have become a research focus in the fields of modern ecology and geography (Alberti et al., 2011). As far as the coupled human and natural system is concerned, there is an ecotone between socio-economic systems and natural ecosystems. It is characterized by high ecological components and strong socio-economic attributes. Its natural and human landscape is different from those of the human and natural systems, and there is a spatiotemporal gradient in the transition between them. As an integrated space where ecological and socio-economic processes are interrelated, some ecotones have uncertain and transitional conditions (Zhang et al., 2022). This kind of ecotone is called an ecological and socio-economic transition zone (Deng et al., 2020). In fact, this term is an extension of the term “ecotone”. It has a transitional nature in terms of its ecosystem (ecological ecotone), industry types (agro-pastoral transition zone or agricultural forestry ecotone), and social and economic development (urban–rural ecotone). The ecological and socio-economic transition zone containing coupled human and natural ecosystems has diverse structures, functionality, and characteristics in terms of its topography, climate, population, land use, and socioeconomic development (Risser, 1995; Shea et al., 2021). Strategically, research on coupled human and natural ecosystems seeks to understand this complexity by integrating knowledge of the constituent subsystems and their interactions. Operationally, this involves connecting sub-models to create coupled models that can accurately reflect human subsystems (such as the economy and society) and natural subsystems (such as the atmosphere, hydrology, and biology), and—most importantly—their interactions (Alberti et al., 2011). While significant progress has been made in this field and many human–nature interactions have been examined (Liu et al., 2007), research into the complexity of coupled systems is limited (Berkes et al., 2008; Smith and Islam, 2019).

Therefore, identifying the ecological and socio-economic transition zone is a necessary step when analyzing its structure, functions, and characteristics, and is also an effective way to explore the complexity of coupled human and natural ecosystems. At present, the best methods are either non-objective or have difficulty identifying complex ecotones (Hufkens et al., 2009a; Dale and Fortin, 2014; Shea et al., 2021). Subjective methods for transition zone detection involve qualitatively determining discrete boundary lines between systems (e.g., natural, rural, or urban boundaries), or delimiting the margins of transition zones at random buffers outside of the boundary lines (Dauber and Wolters, 2004; Urbina-Cardona et al., 2006; Blackwood et al., 2013). This eventually leads to inconsistent reconstruction of transition zones, making it difficult to quantify their characteristics objectively. Objective location determination approaches of transition zones are often based on a specific property, e.g., the turnover or the co-occurrence properties. The turnover property is based on the definition of a transition zone, where the species composition or vegetation structure (also referred to as turnover) changes relatively dramatically (Di Castri and Hansen, 1992; Dale and Fortin, 2014). For example, the extreme values (maximum and minimum) of the second derivative of a sigmoid wave curve fitting can be used to determine the edge of the transition zone using this characteristic (Hufkens et al., 2008; Foster and D'Amato, 2015). This method is suitable when the differences between the systems to be measured are not complex (Hufkens et al., 2009b). When the situation is reversed and the differences between systems are difficult to detect, a method based on co-occurrence properties may be more effective. The fuzzy logic detection method, which reflects regions of highly mixed land-use types, uses this feature (Arnot et al., 2004; Hill et al., 2007). When basic data are supplemented by GIS and RS technology, the transition zone identification method can be applied to large-scale quantitative analyses, such as spatial clustering (Li et al., 2018), wavelets (Camarero et al., 2006), evaluation model construction (Liu et al., 2011), and the moving split-window method (Yu et al., 2015). In addition, change-point analysis is a common method for identifying transition zones, because it can be used to accurately and objectively examine sharp changes in gradients—to a certain extent (Peng et al., 2018). However, there are research gaps for previous studies due to the limitations of the research framework and model. Many frameworks under-recognize the effects of coupled natural and human system. For example, studies on agro-pastoral ecotones are inclined to focus geographical and climatic conditions to the neglect of human activity (Zhang et al., 2009; Qiao et al., 2018). And concern for the economic level, land use and commuting are at the core of the study on urban–rural ecotones (Peng et al., 2018; Feng et al., 2020). The interactive coupling between humans and nature is a complex system involving society, economy, and nature, which involves complex coupling mechanisms. Understanding human- nature interaction is essential to the pursuit of both human wellbeing and global sustainability. To understand the interactions, ecologists, geographers, environmental scientists and other scholars from different disciplines have proposed many research theories and frameworks (Liu et al., 2020), but it is rare in the study of transition zone. These transition zones were only delimitated with spatial boundaries and have specific topographical, climatic, environmental, agricultural, or economic characteristics. Few studies have focused the interaction between ecosystems, especially impacts of the interaction on the spatial boundaries of transition zone. The delineation of a transition zone covering various ecosystem types cannot be achieved in a concise and informative manner. Further, many studies have not considered that regions with transitional characteristics are not large or continuous or large-area form, or that abnormal patches were evident in the transition zone.

Hence, it is imperative to formulate a scientific method to determine the transition zone from the perspective of coupled human and natural ecosystems while comprehensively considering multi-factor characteristics. This model can be used to integrate various social, economic and ecological factors, measure the interaction and direction between subsystems, and accurately delineate ecological and socio-economic transition zones rather than contiguous ones with abnormal patches. This study proposed a conceptual framework of ecological and socio-economic transition zone and structured an index system for transition zone identification. The composite system model was proposed to identify and characterize China’s ecological and socio-economic transition zone. Further, the coupling mechanism of different types of human and natural systems, along with their complexity, were also explored. In general, this study forms a basis for research on coupled human and natural ecosystems by identifying and understanding this specific type of integrated ecotone. It offers further support for regional coordinated development and ecological sustainability policies.

## 2 Materials and methods

### 2.1 Conceptual framework

**Figure 1:**
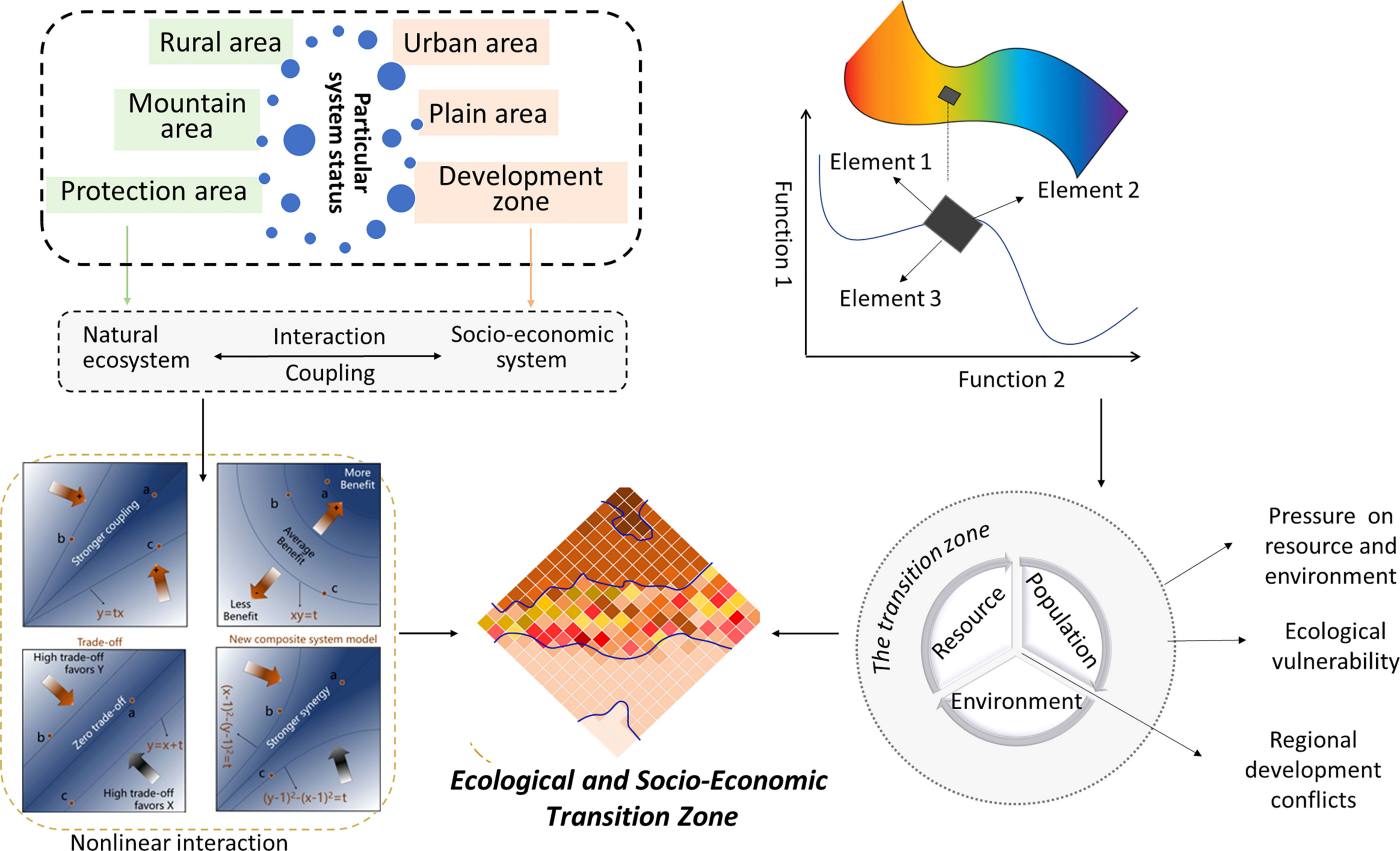
Conceptual framework for ecological and socio-economic transition zone.

Ecological and socio-economic transition zones occur between densely populated urban areas (predominantly affected by human processes) and alpine natural reserves (predominantly affected by natural processes). From the macro-geographical perspective, ecological and socio-economic transition zone is a type of composite geographical spatial entity where humans interact with ecosystems. The theory of spatial ecology has created a new ecological geography paradigm (Peng et al., 2016), which states that this complex ecosystem has a structural hierarchy, versatility, and spatial heterogeneity, in addition to scale dependence (temporal or spatial) and local randomness (Xiao et al., 1997; Chen et al., 2015). Furthermore, its components, structure, and functionality inevitably transform over time under the combined influence of various human and natural factors (Strayer et al., 2003). Further, population–resource–environment problems in such areas are becoming increasingly severe (**Figure 1**).

Generally, there is a negative correlation between human elements and natural elements in a space. However, the complexity of coupled system leads to stronger interactions between humans and nature, in addition to the emergence of ecological and socio-economic transition zone. Based on the aforementioned framework, this study proposes a systematic methodology for identifying the complexity of coupled systems and recognizing the transition zone. It provides coupled models of natural and human subsystems.

### 2.2 Data sources

Remote sensing data, meteorological observation data, soil data, and socioeconomic data were used in this study (**Table 1**). Terra Moderate Resolution Imaging Spectroradiometer NPP data (MOD17A3HGF) and NDVI data (MOD13Q1) were used to characterize agricultural production function of forest land, cultivated land, and grassland. Images were preprocessed using radiometric calibration, atmospheric correction, image mosaics, cloud removal, shadow processing, outlier elimination, and spectral normalization. Climate data—including precipitation and accumulated temperature—were collected from about 2400 meteorological stations. Considering the terrain as an influence, the observation data were interpolated using the smooth spline function in the Australian National University Spline Interpolation Model. Social and economic data were used—including the GDP and added value of agriculture, forestry, animal husbandry, and fisheries—covering 2844 county-level administrative regions in mainland China. The data from these counties were allocated to a 1-km grid to achieve downscaling. Other than DEM and soil data all data were from 2020 and were resampled to 1 km resolution raster data.

**Table 1:** Descriptions and sources of data used in this study.

Data types	Purpose	Sources
Land use data	Soil erosion, Density of settlement patch, Land function	Resource and Environment Science and Data Center (www.resdc.cn). (30-m resolution)
Digital elevation model	Topographic position index, Soil erosion	Shuttle Radar Topography Mission dataset provided terrain data. (90-m resolution)
Normalized difference vegetation index (NDVI) data	NDVI, Forestry production	MOD13Q1 NDVI data was provided by the United States National Aeronautics and Space Administration (NASA) (available online: https://www.earthdata.nasa.gov/eosdis). (250-m resolution).
The net primary productivity (NPP) data	Animal husbandry production, Grain production	MOD17A3HGF NPP data was obtained from NASA. (500-m resolution)
Nighttime light imagery data	Density of GDP	Harvard Dataverse (500-m resolution, https://doi.org/10.7910/DVN/YGIVCD)
Climate data	Humidity index, Accumulated temperature, Soil erosion	The temperature and precipitation data were downloaded from the China Meteorological Data Network. MOD16A2 Evapotranspiration data was obtained from NASA. (500-m resolution)
GDP (by type of industry)	Agricultural production function, Density of GDP	Social and economic data were derived from China Statistical Yearbooks (county-level)
Soil properties	Soil erosion	Harmonized World Soil Database.
Protected area data	Natural property index	The World Database on Protected Areas
Road data	Traffic accessibility	Open Street Map

### 2.3 Model construction

#### 2.3.1 Index system and quantitative method

The index system used for identifying ecological and socio-economic transition zones was established according to several criteria (**Table 2**), with consideration of the crucial issues and the characteristics of China (Danz et al., 2011; Chen, 2018; Wang and Li, 2019; Zhang, 2019; Liu et al., 2021a; Sun et al., 2021), In this study, natural property indexes were divided into five factors: landform, hydrology, temperature, soil, and vegetation. First, protected areas and areas at altitudes > 4500 m were judged to be in a purely natural ecological state. Natural conditions such as terrain and landform greatly affect the form and structure of the landscape and ecosystem (Feng et al., 2008). The terrain position index can be used to evaluate terrains and landforms, and a single index can reflect both slope and altitude information. The amount of water resources in a region significantly affects soil quality, agricultural irrigation, and vegetation growth, which are crucial for maintaining the basic living environment in a region (Li and Li, 2012). In addition, inclement weather and vegetation cover are associated with unpopulated areas with strong natural properties (Li et al., 2021). The humidity index and accumulated temperature can affect regional water and heat resources, and are also a reflection of the climate. Vegetation cover can reflect the intensity of human activities and the overall condition of the natural environment, and is expressed by the NDVI. Erosion degrades the soil quality in natural systems and land reduces productivity. This leads to a diminished diversity of plants, animals, and microbes, as well as a harsh natural environment (Pimentel and Kounang, 1998).

**Table 2:** Index system for identifying ecological and socio-economic transition zones in China.

Target layer	Criteria layer	Index layer	Sub-index layer
Composite system index	Natural property index	Landform	Topographic position index (TPI)
		Hydrology	Humidity index (HI)
		Temperature	Accumulated temperature (≥10°C) (AT)
		Soil	Soil erosion (SE)
		Vegetation	Normalized difference vegetation index (NDVI)
	Human attribute index	Population	Density of settlement patch (DSP)
		Land function	Agricultural production function (APF)
		Economy	Density of GDP
		Traffic location	Traffic accessibility (TA)

The human attribute index is reflected by four factors: population, land use, economy, and traffic location. Built-up land—a factor of the land use type—is regarded to be a pure social economy state because of its overly strong human attributes. The GDP density and density of settlement patches were used to embody the intensity of human activity, the spatial pattern of urban construction, and the level of urbanization; these can better signify the primary characteristics of human elements (Peng et al., 2019). The agricultural production function reflects the extensive anthropogenic influence of land use (Fan et al., 2021). Owing to agricultural production (agriculture, forestry, animal husbandry, and fisheries), human–environment interactions have experienced major changes. Furthermore, there has been a loss of area with strong natural properties, thereby the human attributes have been enhanced in these areas. Agriculture is the world’s leading type of land use, and has far-reaching impacts on biodiversity and ecosystem services (Plieninger et al., 2016). Traffic accessibility is an important and indispensable factor in estimating the intensity of human attributes (Jin et al., 2010). Detailed information about the index quantification methods is shown in **Table 3**. Index values were normalized using the extremum difference method, and weights were determined by combining subjective and objective methods (analytic hierarchy process and entropy weight method). It is worth noting that the NDVI, humidity index, and accumulated temperature are negative indicators (the smaller the value, the stronger natural properties) at the national scale.

**Table 3:** Quantification methods for natural property and human attribute indexes used in this study.

Index	Calculation process
Topographic position index (TPI)	$TPI_{i}=Lg\left[\left(\frac{E_{i}}{\bar{E}}+1\right)\times \left(\frac{L_{i}}{\bar{L}}+1\right)\right]$ *TPI_i_* is topographic position index of unit *i*; *E*_i_ and *L_i_* are the elevation and slope of unit *i* in the study area, respectively; and $\bar{E}$ and $\bar{L}$ are the average elevation and average slope in the area, respectively (Zhou et al., 2021).
Humidity index (HI)	$HI=\frac{P}{ET_{0}}$ *P* is annual mean precipitation, and *ET_0_* is potential evapotranspiration (He et al., 2011).
Accumulated temperature (≥10°C) (AT)	It is obtained by summing all daily average temperatures greater than 10°C in a year
Soil erosion (SE)	*SE*=*R*·*K*·*LS*·*C*·*P* *SER*, *K*, *LS*, *C*, and *P* are the rainfall erosivity, soil erodibility factor, slope length-gradient factor, crop management factor, and support practice factor, respectively (Keller et al., 2015; Yao et al., 2016).
Normalized difference Vegetation index (NDVI)	Obtained using the maximum value composite method after pretreatment (Peng et al., 2022).
Density of settlement patch (DSP)	DSP is acquired by calculating the aggregation degree of urban land and rural settlements in land use types (Fan et al., 2021).
Agricultural production function (APF)	Obtained by measuring the agriculture production capacity at the grid scale combining statistical data, NPP, NDVI, and land use data (Sun et al., 2017; Hong et al., 2019; Li et al., 2020; Chen et al., 2022). The added values of agriculture, forestry, animal husbandry, and the fishery industry were used to unify and quantify their production capabilities.
Density of GDP	Estimate the density of GDP at the grid scale, combining statistical data at the county level and nighttime light imagery data (Han et al., 2012).
Traffic accessibility (TA)	Superimpose road network density at all levels, with traffic efficiency as the weight (Jin et al., 2010).

#### 2.3.2 The model for measuring the interactions between two systems

The coupling degree (*CP*), derived from physics, is a representation of the degree of correlation between *n* systems or between *n* elements within a system (Maimaiti et al., 2022). The coordination degree (*CD)* was used to reflect the overall relationship between them, in addition to the relevance, considering the weight of each objective (*w_i_*) (Zhang et al., 2016). The trade-off (*TO*) between *n* objectives can be reflected by the root mean square error (*RMSE*) (Peng et al., 2019).


$$CP=n\sqrt[{n}]{\frac{\prod_{i=1}^{n}b_{i}}{{\left(\sum_{i=1}^{n}b_{i}\right)}^{n}}}$$

$$CD=\sqrt{CP\times \left(\sum_{i=1}^{n}b_{i}\cdot \omega_{i}\right)}$$

$$TO=RMSE=\sqrt{\frac{1}{n-1}\sum_{i=1}^{n}{\left(b_{i}-\bar{u}\right)}^{2}}$$

Where *b_i_* is the benefit of objective *i* and $\bar{u}$ is the mean benefit (expected value) for objective *i*. The *RMSE* is an approximation of the average deviation of the mean benefit. In two dimensions, $\bar{u}$ is on the zero trade-off line. The *TO* for two objectives is defined as the distance from the zero trade-off line (Peng et al., 2019). A larger *RMSE* implies a higher trade-off between the two, and vice versa.

**Figure 2:**
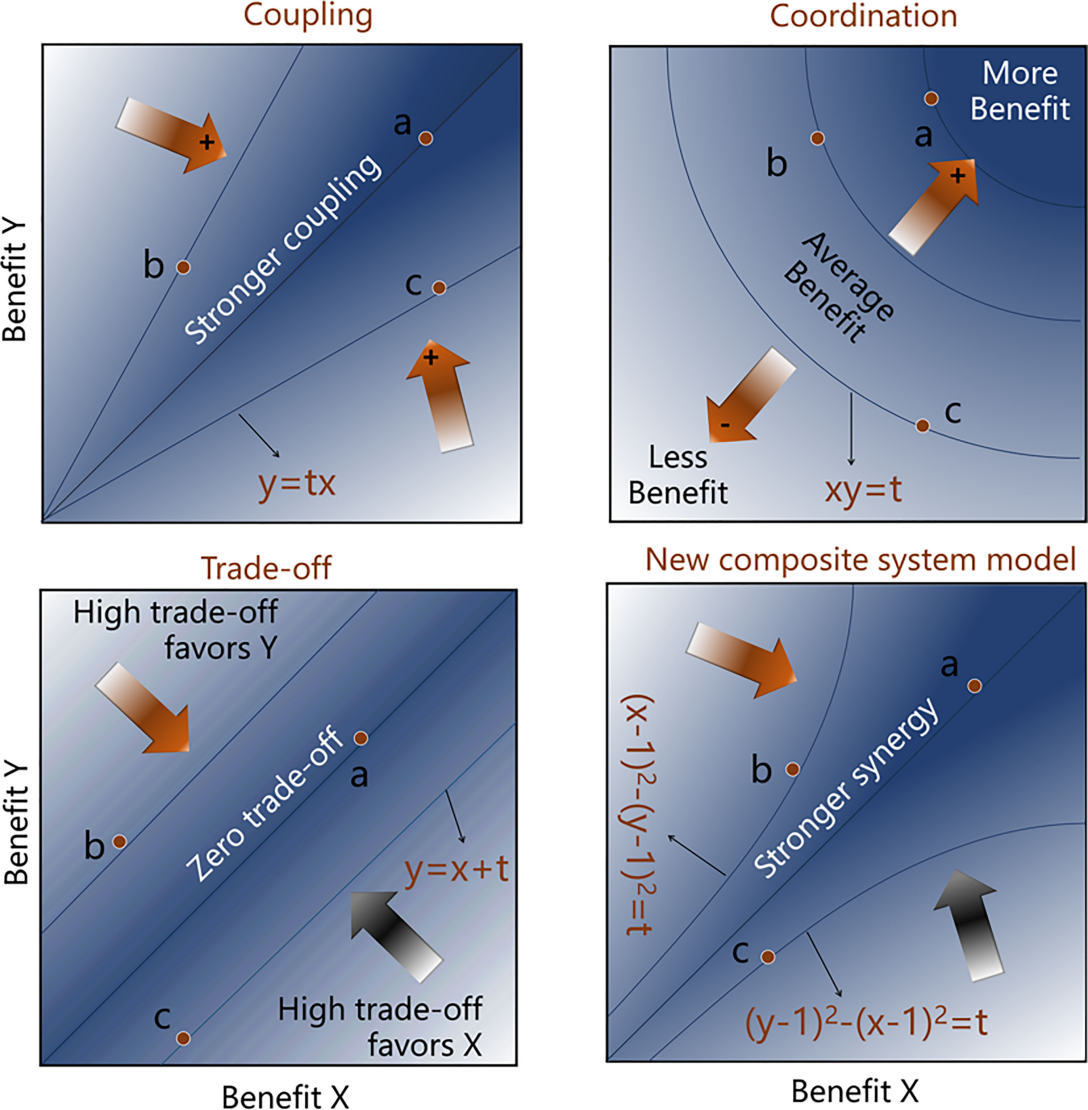
Schematic diagram of coupling, coordination, trade-offs, and composite system index (CSI) between benefits of two objectives. The order for the coupling, coordination, trade-off, and CSI of dots a, b and c is |a| > |b| = |c|. Among them, b = -c can only be calculated in the trade-off model and the new composite system model. The letter *t* stands for an uncertain parameter.

We improved the traditional coupling coordination model to be able to determine the coupling degree (the interaction), comprehensive level, and orientation of interactions between the two systems. The new composite system model was obtained through the superposition of the trade-off model and coordination model. The smaller the result of the trade-off model, the better the system state. The coordination model exhibits the opposite of this phenomenon, so it is necessary to negatively transform part of the new model. Based on the derived formula, we have given the calculation process of the composite system index (CSI), along with a schematic diagram (**Figure 2**).


$$\begin{array}{l} CSI=\sqrt{\left(\sqrt{\frac{1}{n-1}\sum_{i=1}^{n}{\left(b_{i}-\bar{u}\right)}^{2}}\right)\times \left\{z^{+}+z^{-}-\left(\sum_{i=0}^{n}b_{i}\cdot \omega_{i}\right)\right\}}\\ z^{+}=\max\left(\sum_{i=0}^{n}b_{i}\cdot \omega_{i}\right)\\ z^{-}=\max\left(\sum_{i=0}^{n}b_{i}\cdot \omega_{i}\right) \end{array}$$

$$b_{i}\in \left[0,1\right]$$

In this study, human systems (x) and natural systems (y) are considered, and both have equal importance. To identify the orientations of the interactions between systems, the positive and negative signs are retained throughout the calculation process (the positive and negative scenarios are discussed separately). When *n*=2, *w_1_* = *w_2,_ x* > *y*; therefore,


$$\begin{array}{l} CSI=\sqrt{\sqrt{\left\{{\left(x-\frac{x+y}{2}\right)}^{2}+{\left(y-\frac{x+y}{2}\right)}^{2}\right\}}\times \left\{2-\left(x+y\right)\right\}}\\ CSI=\sqrt{\frac{\left(x-y\right)}{\sqrt{2}}\times \left\{2-\left(x+y\right)\right\}}=\sqrt{\frac{2x-2y-x^{2}+y^{2}}{\sqrt{2}}}=\sqrt{\frac{{\left(y-1\right)}^{2}-{\left(x-1\right)}^{2}}{\sqrt{2}}} \end{array}$$

x,y∈[0,1]

Similarly, the case where x is less than y can be deduced. The final formula is


$$2\cdot CSI^{2}=\left\{ \begin{array}{l} {\left(y-1\right)}^{2}-{\left(x-1\right)}^{2},\left(x>y,CSI>0\right)\\ {\left(x-1\right)}^{2}-{\left(y-1\right)}^{2},\left(x<y,CSI<0\right)\\ y-x,\left(x=y,CSI=0\right) \end{array}\right.$$

x,y∈[0,1]

## 3 Results

### 3.1 Human attributes and natural properties

**Figure 3:**
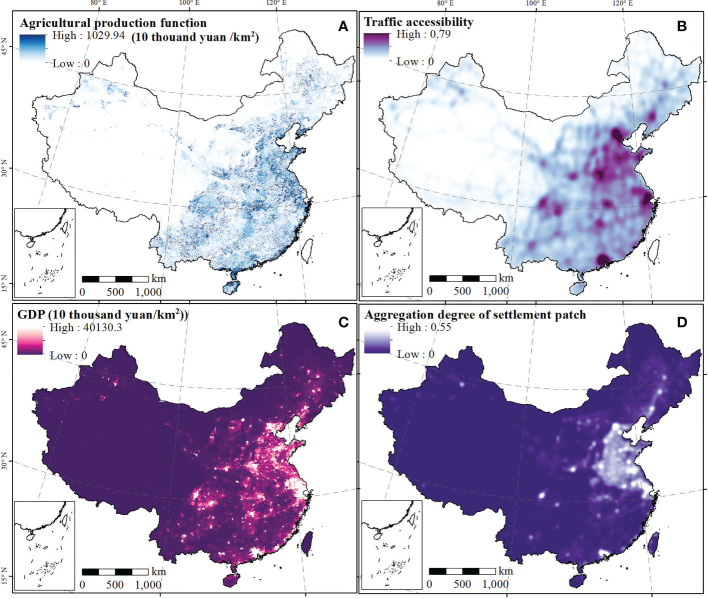
The human attributes across China. Maps **(A-D)** represent the spatial distribution of agricultural production function, traffic accessibility, GDP, and aggregation degree of settlement patch, respectively.

**Figure 4:**
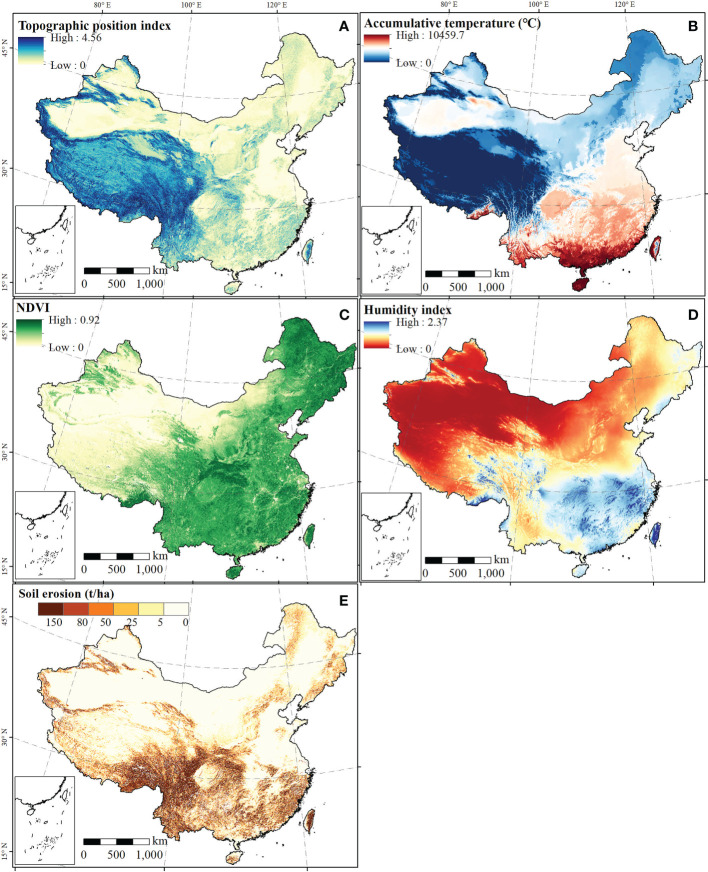
The natural properties across China. Maps **(A-E)** represent the spatial distribution of topographic position index, accumulative temperature, NDVI, Humidity index, and soil erosion, respectively.

The spatial distribution of human attributes (socio economic level) over China exhibits an increase from the northwest to the southeast, as shown in **Figure 3A**. For example, the regions with the highest agricultural production function (> 10 million yuan/km^2^) were in Central China and the coastal provinces (**Figure 3A**). traffic accessibility is affected by roads and presented a linear aggregated distribution (**Figure 3B**). The traffic accessibility values in the southeast half of China had the highest values, and significantly outperformed the northwest. Moreover, the highest GDP could be found in Beijing-Tianjin-Hebei Urban Agglomeration, Yangtze River Delta Urban Agglomeration, Pearl River Delta Urban Agglomeration, Chengdu-Chongqing Urban Agglomeration, and the Triangle of Central China, and the highest value was more than 40 million yuan/km^2^ (**Figure 3C**). The high values of aggregation degree of the settlement patch were primarily distributed in contiguous areas of Beijing, Tianjin, Hebei, Shandong, Henan, Jiangsu, Shanghai, and northern Anhui (**Figure 3D**). A high value of the topographic position index was found in the Tibetan Plateau and Tianshan Mountains, and a low value was found in the North China Plain, Northeast Plain of China, and Tarim Basin, with values ranging from 0 to 4.56 (**Figure 4A**). It can be seen in **Figure 4** that the accumulated temperature, humidity index, and NDVI in southern China was higher than that in northern China, and that in western China was lower than that in eastern China. Soil erosion in China is divided into five grades according to Chinese national standards, and high erosion regions were mainly found in the southeast Tibetan Plateau, the Yunnan-Guizhou Plateau, and low mountains and hills in Southeast and South China (**Figure 4E**).

**Figure 5:**
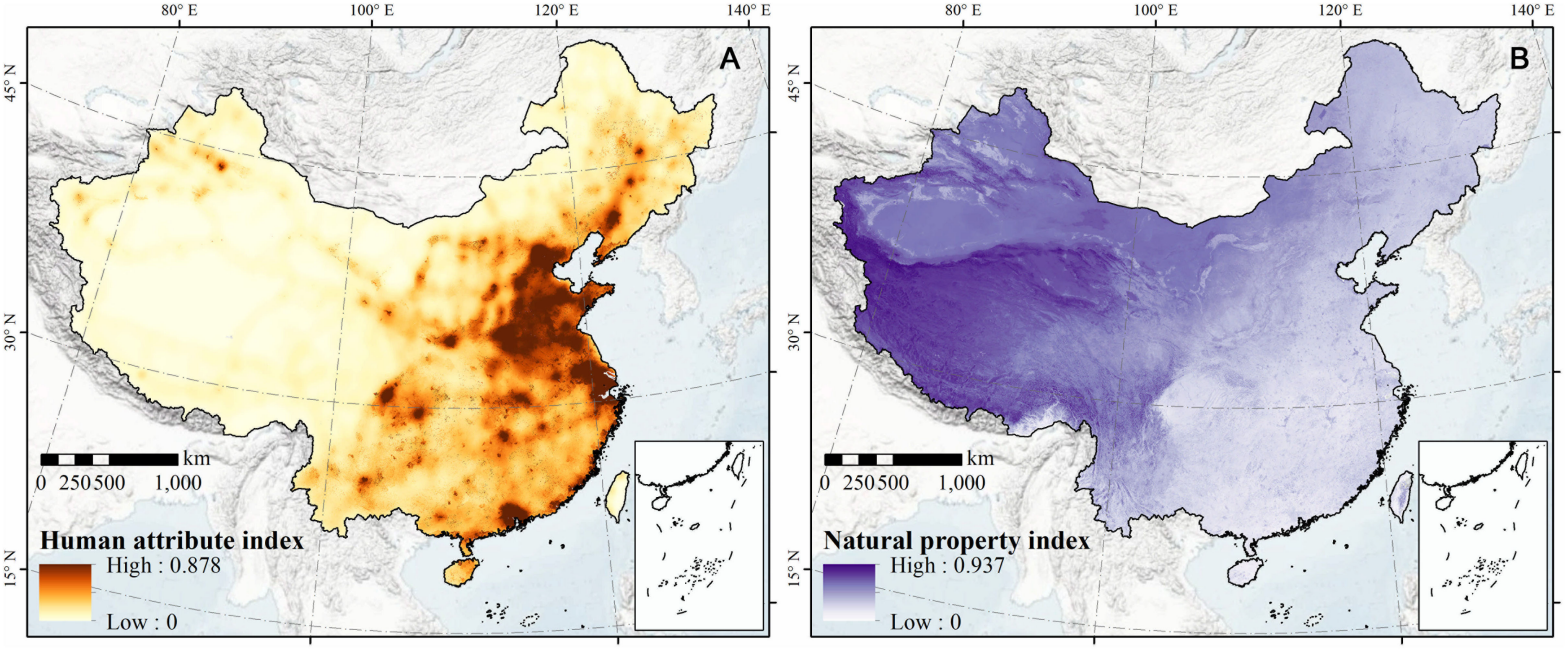
The human attribute index **(A)** and natural property index **(B)** across China.

**Figure 5A** shows the human attribute index in China, which reflects socio-economic development and the intensity of human activities. **Figure 5B** reveals the distribution of the natural property index, which is affected by natural elements (Landform, soil, vegetation, and climatic conditions). They were acquired through the weighted superposition (analytic hierarchy process) of standardized population, economy, land function, and traffic location. Overall, the human attribute index of the northwest region was lower than that of the southeast region, exhibiting a downward trend from urban agglomeration areas to natural ecological areas (with high altitudes). The human attribute indexes of the seven major urban agglomerations in China—including the Beijing-Tianjin-Hebei Urban Agglomeration, Yangtze River Delta Urban Agglomeration, Pearl River Delta Urban Agglomeration, Chengdu-Chongqing Urban Agglomeration, Central Henan Urban Agglomeration, Guanzhong Plain Urban Agglomeration, and Triangle of Central China, were higher than those of other regions. **Figure 5B** shows the distribution of the natural property index, which is affected by natural ecological elements (the landform, soil, vegetation, and climatic conditions). In general, the poorer the geographical environment, the more unfavorable the climatic conditions and the higher the natural property index. The spatial pattern shows that the natural property index in the northwest was higher than that in the southeast, and the natural property index in the middle and high mountains was higher than that in the plains and hills of China. Specifically, the Tibetan Plateau, Inner Mongolia Plateau, Loess Plateau, and Xinjiang (in western China), which had higher mountains, steeper slopes, or arid areas, exhibited stronger natural ecological properties. On the other hand, plain and hilly areas, which were in southeast China and the Sichuan Basin, were primarily dominated by extremely weak natural properties due to their ideal terrains and climatic conditions.

### 3.2 Coupled human and natural ecosystems

**Figure 6:**
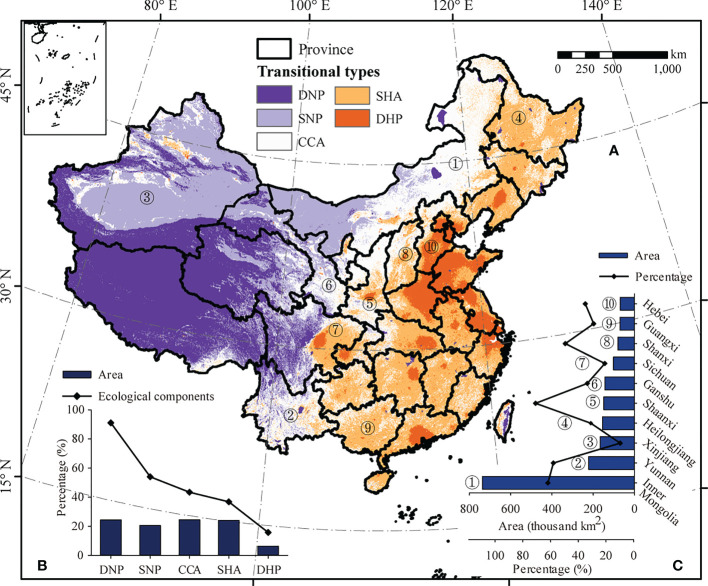
Composite system index across China **(A)**, proportion of each type and its ecological components **(B)**, and 10 provinces/regions with the largest complex coupling area proportion **(C)**. Areas dominated by human attributes, areas with strong human attribute-low natural properties, complex coupling areas, areas with strong natural property-low human attributes, and areas dominated by natural properties are represented by DHP, SHA, CCA, SNP, and DNP, respectively.

The coupling coordination model and the trade-off model were fused into a new composite system model. Further, the composite system index (CSI) was created, which characterizes the coupling degree, coordination degree, and direction of interaction between the human and natural systems in China. A CSI > 0 indicates that human attributes are stronger than natural properties, and vice versa. A CSI close to 0 indicates that the two have a complex relationship and similar effects. It can be seen that the lower (higher) the CSI, the stronger (weaker) the natural properties, and the more (less) the natural ecological components, indicating a weaker coupling coordination degree of the nature–human system. When the CSI reaches its highest value, the ecological components are 0%. Furthermore, protected areas and areas at altitudes greater than 4500 m are considered to be areas with absolute natural properties (100% ecological components), and built-up land is considered to be an area with absolute human attributes (0% ecological components). Based on the principle of the minimum difference within groups and maximum difference between groups, the degree of interaction of nature–human systems (composite system index) in China was graded into five groups (**Figure 6A**), namely areas dominated by the human process (DHP), areas for strong human attribute-weak natural properties (SHA), complex coupling areas (CCA, i.e. ecological and socio-economic transition zone), areas with strong natural property-weak human attributes (SNP), and areas dominated by natural processes (DNP). The CCA is considered as ecological and socio-economic transition zone, accounting for 24.56% of the total area (**Figure 6B**).

The CSI in China exhibits the following overall characteristics: the DNP mainly emerged in the Tibetan plateau, the DHP mainly appeared in the North China Plain, and the CCA presented a banded distribution between the DNP and the DHP. The SHA, the CCA, and the SNP were transition regions between the strong natural property area and the strong human attribute area. Further, the proportion of the area occupied by these five types was counted, and ordered from low to high: NHC (6.38%), SNP (20.61), SHA (24.1%), DNP (24.35%), and CCA (24.56%) (**Figure 6B**). In addition, the ecological component of each type of area was calculated, and the stronger the ecological properties, the higher the ecological component. The ecological component of DNP reached as high as 91.06%, and the DHP—with the lowest ecological component—had a value of 15.8%. The ecological component of CCA was 43.43%. The top ten provinces/regions with the largest area of CCA are listed in **Figure 6C**. The top ten provinces/regions account for 78.5% of the CCA in China.

**Figure 7:**
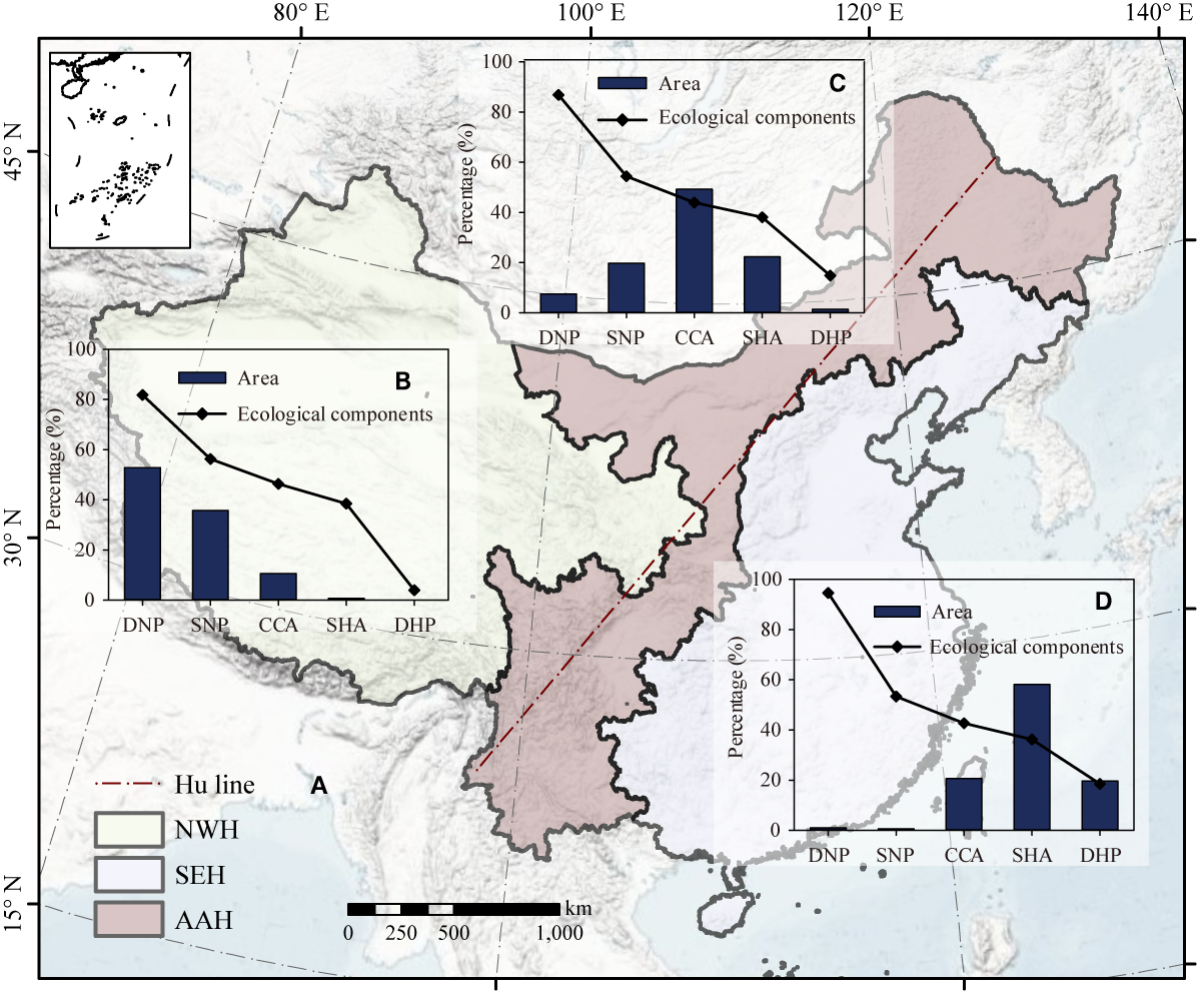
Zoning based on the Hu line in China **(A)**. The proportion of each type and its ecological components northwest of the Hu line **(B)**, the area across the Hu line **(C)**, and southeast of the Hu line **(D)**, along with the proportion of each type **(B)**. 10 provinces/regions with the largest complex coupling areas **(C)**. Areas dominated by human attributes, areas with strong human attribute-low natural properties, complex coupling areas, areas with strong natural property-low human attributes, and areas dominated by natural properties are represented by DHP, SHA, CCA, SNP, and DNP, respectively. Additionally, southeast of the Hu line, the area across the Hu line, and northwest of the Hu line are represented by SEH, AAH, and NWH, respectively.

To further analyze the distribution of ecosystem types and the characteristics of the ecological components in each region, China was divided into three regions based on provincial boundaries (**Figure 7A**): northwest of the Hu line (NWH), the area across the Hu line (AAH), and southeast of the Hu line (SEH). The area of DHP, SHA, CCA, SNP, and DNP in NWH increased sequentially (**Figure 7B**). The area of SNP and DHP accounted for 88.58% of the region. The histogram of the area proportion of the five ecosystem types in AAH had a pyramid shape, and the area of CCA accounted for half (49.24%) of the total area (**Figure 7C**). The largest area in SEH was the that of SHA, accounting for 58.11% of the total (**Figure 7D**).

The ecological components all over China exhibited an increase from the southeast to the northwest, and the same pattern was observed for the ecological components in the areas of NHC, CCA, and SHA. However, an opposite trend in ecological components was found in DNP and DHP, that is, a decrease from the southeast to the northwest. The DHP areas in NWH region were almost all composed of built-up land, while the DNP areas in SEH are almost all protected. Hence, they had either completely human attributes or natural properties.

### 3.3 Control factors of CSI in the entire region and certain subdivisions

**Figure 8:**
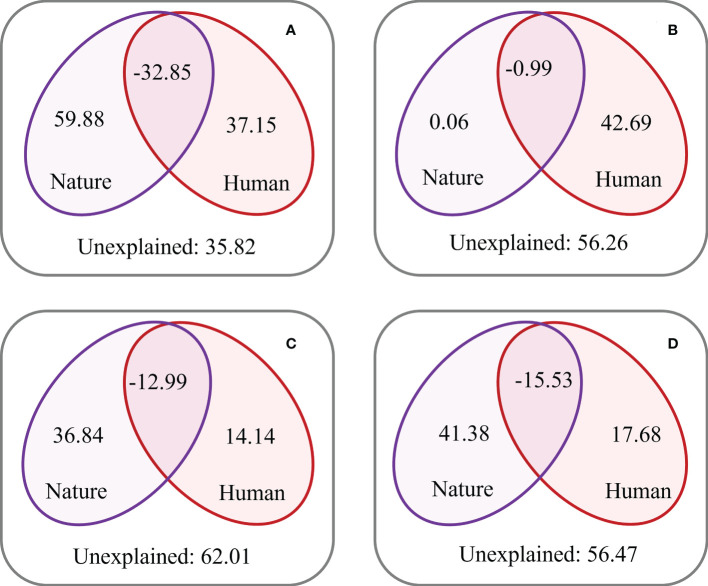
Relative contributions of natural properties and human attributes to the composite system index (CSI) in China **(A)**, southeast of the Hu line **(B)**, the area across the Hu line **(C)**, and northwest of the Hu line **(D)**.

Variation partitioning analysis indicated that, in China, AAH and NWH (**Figures 8A, C, D**), along with natural properties (59.88%, 36.84%, and 41.38%), explain a larger proportion of the variance in CSI than human attributes (37.15%, 14.14%, and 17.68%). Variation partitioning analysis also indicates that, in SEH (**Figure 8B**), human attributes (42.69%) explained a greater proportion (0.06%) of the variance in CSI than natural properties. Human attributes and natural properties—in combination—explained 37.99% of the CSI variation (**Figure 8C**), while the unexplained proportions were as high as 62.01% in the AAH, which is greater than those for China (35.82%), and the SEH (56.26) and NWH regions (56.47%).

**Figure 9:**
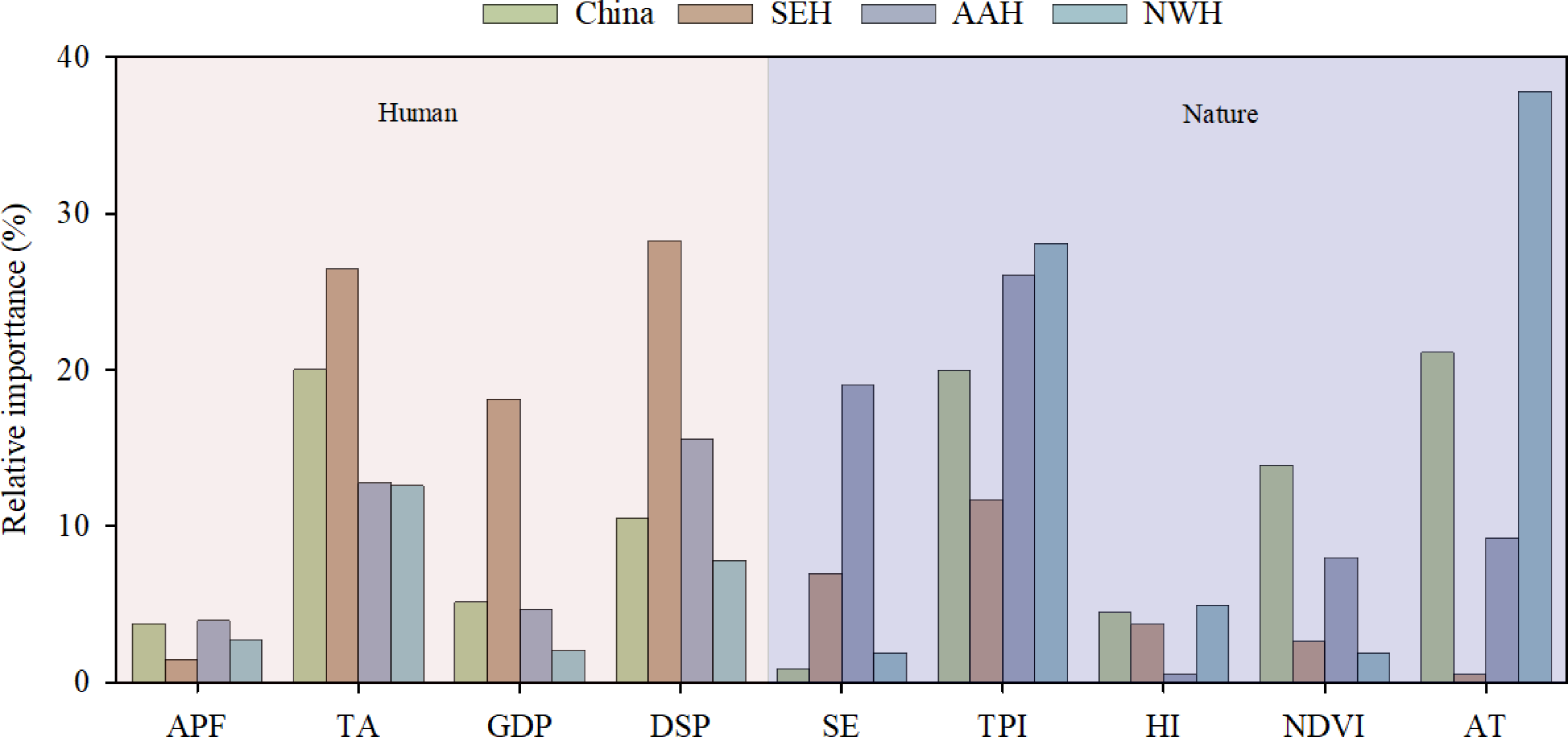
Relative importance of natural and human elements explaining the composite system index (CSI). Natural and human elements include the agricultural production function (APF), traffic accessibility (TA), GDP, density of settlement patches (DSP), soil erosion (SE), topographic position index (TPI), humidity index (HI), normalized difference vegetation index (NDVI), Accumulated temperature (≥10°C) (AT). Furthermore, southeast of the Hu line, the area across the Hu line, and northwest of the Hu line are represented by SEH, AAH, and NWH, respectively.

The relative importance of nine natural and human elements for CSI was further analyzed (**Figure 9**). The results indicated that the AT was the critical element associated with CSI in the NWH region, accounting for 37.81% of the CSI variation. Additionally, the TPI (26.05%) was the critical element in AAH, and the DSP (28.29%) was the critical element in SEH. Nationwide, the AAT (21.13%), TA (20.03%), and TPI (19.98%) were the three most important elements, but their proportions were lower than in localized areas. In general, the CSI variation in NWH was dominated by natural elements, that in SEH by human elements, and that in AAH by both.

## 4 Discussion

### 4.1 Natural and human features in ecological and socio-economic transition zone

Many studies have found that there are transition areas or ecologically sensitive areas near the Hu line, with significant gradients in terms of the climate, topography, and population (Hu et al., 2015; Qi et al., 2016; Chen et al., 2016; Liu et al., 2021a). The ecological component of CCA was 43.43%, which was consistent with the prior conjecture for ecological and socio-economic transition zone (Deng et al., 2020). This is the transition zone that the research aimed to identify.

In the light of the planning of the main functional area of China (2011), the per capita water resources in ecological and socio-economic transition zones are relatively scarce and many key eco-functional areas and ecologically fragile areas are concentrated nearby. Further, the biophysical boundary conditions, climate change, and the over-exploitation of resources have much greater adverse impacts on ecosystems in dryland than in non-dryland areas (Adeel et al., 2005). Large areas of dryland are distributed in ecological and socio-economic transition zones in China, where climate change and water shortages restrict land development and utilization (Wang and Li, 2019). Furthermore, the resources in the transition zones are more diversified; however, the environment is more fragile due to edge effects and a poor resource mix. Therefore, a large proportion of counties with low economic development and relatively poor rural populations (formerly poor populations) are distributed in the transition zones, primarily in areas with insufficient natural resources and poor geographical conditions, such as Inner Mongolia, Ningxia, Shaanxi, Gansu, Yunnan, and Sichuan, which can easily fall into the trap of “green poverty” (Wang and Li, 2019).

The proportion of areas with stronger natural properties in SEH was significantly small; most of those areas were situated on the third step of China’s terrain, with a prosperous economy, denser population, and an altitude of less than 500 m (**Figures 7A, D**). In comparison to the SEH, the severe environment of the NWH have less urban land (Chen et al., 2019) (**Figures 7A, B**). The ecological components of DHP in NWH were lower than those in SEH, because they are almost all composed of built-up land. The ecological components of DNP in SEH were higher than those in NWH, because the former is primarily composed of protected areas and has strong natural properties. In some mountainous areas of the AAH, including the Yunnan-Guizhou Plateau, Heng-duan mountains, Changbai Mountains, and the nearby Xing ‘an Mountainous Region, the proportion of areas with strong human attributes was also very low (Liu et al., 2021a) (**Figures 7A, C**).

Ecological and socio-economic transition zones can serve as an ecological barrier between the east and west of China (more specifically, between the human system natural system) for water conservation and desertification prevention; however, it suffers from ecological environmental destruction when human intervention becomes severe. In particular, in the transition zone of the upper reaches of the Yangtze River and the middle and upper reaches of the Yellow River, soil erosion, land degradation, and geological disasters often occur due to excessive reclamation, grazing, and logging. The ecological restoration and land management in ecological and socio-economic transition zones form a foundation and guarantee for ecological security and sustainable development in economically developed plain areas of the Yellow River and Yangtze River Basins (Wang and Deng, 2016). It is clear that the prioritization of economic development is unsuitable for the transition zones. There is a reason why the main payments for environmental service projects (including the Green Food Programme) are concentrated in the transition zones. These projects promoted the coordination of human–land relations and the construction of an ecological civilization (Zhou et al., 2019; Xu et al., 2020; Liu et al., 2021b) by effectively changing the land use mode, which moderately increases the agricultural income (Yin et al., 2014).

### 4.2 Complexity of the ecological and socio-economic transition zone

As the core coupling region of human and natural systems, ecological and socio-economic transition zones represent transitions from natural systems to human systems, where individual elements are organized into interacting and interdependent subsystems at multiple scales (Chen et al., 2015). This field of research provides a significant framework for understanding the complex interactions between natural and human changes (Ostrom, 2009; Gatzweiler, 2014; Arnaiz-Schmitz et al., 2018). Nearly half of the AAH was an ecological and socio-economic transition zone, which covers a large number of complex coupling areas. Variation partitioning analysis indicated that human attributes and natural properties, in combination, explained only 37.99% of the variation in the CSI. The unexplained proportion in the AAH (62.01%) was greater than that in the SEH (43.74%) and NWH (43.53%; **Figure 8**). This indicates that the variations in complex coupling areas were difficult to interpret. Specific socio-economic and ecological processes produce intricate interfaces, which are reflected in the landscape and internal configuration of ecosystems, and involve their relationships with the surrounding environment (Vizzari et al., 2018). As a result, the multifaceted interactions between the evolving environment and social economy are clear. Nationwide, the TPI was an important element associated with CSI, and accounted for nearly 20% of the CSI variation (**Figure 9**). Among the nine factors the TPI has the highest proportion in the AAH accounting for 26.05%. This is because both topography and landforms are the fundamental components of multi-scale geographical patterns, and directly determine the spatial configuration and landscape types of natural and human elements on the surface (Melelli et al., 2017). In comparison to the NWH and SEH, the CSI variation in the AAH was dominated by both natural and human elements. Therefore, it is clear that the transition zones are complex systems comprised of coupled human and natural elements.

A ecological and socio-economic transition zone is not a single natural ecosystem or urban environment, and differs from an environment dominated by human or natural processes (Vejre et al., 2010). From the perspective of the human activity mode and intensity, an ecological and socio-economic transition zone has uncertainty and complexity in terms of human activities, where livelihoods are influenced by climate change, urbanization, government policies, etc. (Liu et al., 2007). In a traditional agricultural society, especially in mountainous areas, arable land is subject to more limitations, and the land is primarily used for agricultural production (Wang et al., 2016). In the modern industrialized and urbanized society, the combination of market towns, enterprise construction, and the tourism and leisure industry has led to several simultaneous land-bearing functions, and the livelihoods of residents have become diversified (primary, secondary, and tertiary industries). The transition zone has new land intensions and attributes of modern society, and is the synthetic embodiment of production–living–ecological function, with certain trade-offs and synergies (Zou et al., 2021). The ecosystem, population, resources, and environment of an ecological and socio-economic transition zone have special interactions and spatiotemporal evolution characteristics. Ecological and socio-economic transition zones are a focus of ecological and geographical research. Such research provides detailed information on coupled human and natural ecosystems, and reflects the distinctiveness of interactive social economy and environment (Liu et al., 2021a).

### 4.3 Methodology applicability

Ecological and socio-economic transition zones have strong interaction processes, high degrees of spatial heterogeneity, and significant cascading effects. Therefore, it is particularly important to seek a particular tool for governance and developmental policies to deepen our understanding of the spatio-temporal evolution mechanism of the transition zones to better implement regional development decisions and ecological management.

The general identification methods of transitional zone such as fuzzy logic and clustering techniques based on spatial raster have proved to be handy and lead to a more flexible delineation, while they are sensitive to the image noises, and more suitable for a small-scale detailed description. Moreover, these methods were used to directly implement the transitional zone identification (Liu et al., 2021a). To ensure applicability to complex coupled natural–human systems, these methods (based on the superposition of environment factors) need to consider human influences and their interactions. The CSI is such a feasible research tool that, represents the strength of human–nature interactions. It can quantify the interactions within coupled human–natural system, measure their directions, and be used to delineate transition zones. Our study first separately superimposed the ecological and social-economic elements, measured the interaction between human and natural system, and then conducted the complex coupling areas (i.e., ecological and socio-economic transition zone) identification. We used raster data including climate, soil, landform and vegetation and statistical data including population density, economic density, and added value of agriculture, forestry, animal husbandry and fishery. The functions of planting, forestry and animal husbandry have a strong correlation with NPP or NDVI, through which agricultural production functions can be spatialized. We not only considered more comprehensive indicators and multi-source data, but also analyzed the interaction (coupling degree and coupling direction) of the two systems to identify the ecological and socio-economic transition zone. Additionally, the appropriate classification of the CSI could be used to identify various types of transition zone. For example, areas with a positive and high index (e.g., the SHA) may be urban–rural ecotones, and those with negative and low indexes (e.g., the SNP) may be agro-pastoral ecotones. This is a synthetic and systematic index for identifying transition zones that is worthy of further study.

## 5 Conclusions

Ecological and socio-economic transition zone was described by the composite system model according to the intensity and direction of the coupling between its natural properties and human attributes. They exhibited a high degree of transition, complexity, and sensitivity. The CSI is a systematic way to identify the ecological and socio-economic transition zone, and may be able to delimit urban–rural ecotones, mountain–plain transition zones, and agro-pastoral ecotones. The transition zones defined in this study accounted for approximately 1/4 of China’s total land area. The sustainable development of the transition zones faces many challenges, such as complex spatial trade-offs and the maintenance of healthy ecosystems. Therefore, it is imperative to strengthen the in-depth analysis of this type of ecosystem and obtain more detailed knowledge of the mechanisms of interaction between humans and nature.

## Data availability statement

The raw data supporting the conclusions of this article will be made available by the authors, without undue reservation.

## Author contributions

Conceptualization, HZ and FL. Methodology, HZ. Software, JZ. Validation, JZ. Resources, FL. Data curation, HZ. Writing—original draft preparation, HZ. Writing—review and editing, FL. All authors have read and agreed to the published version of the manuscript.

## Funding

This research was funded by the National Natural Science Foundation of China (Youth Program, grant number 42101215) and Western China Young Scholars Program of the Chinese Academy of Sciences.

## Conflict of interest

The authors declare that the research was conducted in the absence of any commercial or financial relationships that could be construed as a potential conflict of interest.

## Publisher’s note

All claims expressed in this article are solely those of the authors and do not necessarily represent those of their affiliated organizations, or those of the publisher, the editors and the reviewers. Any product that may be evaluated in this article, or claim that may be made by its manufacturer, is not guaranteed or endorsed by the publisher.
